# Utilization of Imaging Modalities in the Diagnosis of Melanoma: A Scoping Review

**DOI:** 10.7759/cureus.54058

**Published:** 2024-02-12

**Authors:** Lucas Shapiro, Mahi Basra, Hemangi Patel, Collin Payne, Brett Brazen, Alejandro Biglione

**Affiliations:** 1 Osteopathic Medicine, Nova Southeastern University Dr. Kiran C. Patel College of Osteopathic Medicine, Clearwater, USA; 2 Sports Medicine, Nova Southeastern University Dr. Kiran C. Patel College of Osteopathic Medicine, Fort Lauderdale, USA; 3 Dermatology, Broward Health, Fort Lauderdale, USA; 4 Internal Medicine, Wellington Regional Medical Center, Wellington, USA

**Keywords:** sentinel node biopsy, ultrasound imaging, dermoscopy image analysis, reflectance confocal microscopy, research in melanoma

## Abstract

Melanomas arise de novo or in the context of a precursor lesion. Lesions typically grow radially and then undergo a vertical growth phase proceeding to local invasion and metastasis. This review describes the utility of different imaging modalities in diagnosis and melanocytic lesion monitoring. A literature search was performed in November 2023 utilizing EMBASE, Medline, and PubMed. The PRISMA diagram demonstrates the review process. Reflectance confocal microscopy (RCM) utilizes near-infrared light to help diagnose dermatologic lesions. RCM was found to demonstrate nearly two times the positive predictive value compared to dermoscopy. The introduction of the Berlin Ultrasound (US) Morphology Criteria permitted a 65-80% improvement in diagnostic sensitivity. US with fine-needle aspiration cytology (FNAC) accurately predicts the necessity for sentinel lymph node biopsy and lymphadenectomy, sparing patients with metastasis and prompting biopsy for equivocal lesions. Single-photon emission computed tomography/computed tomography (SPECT/CT) is an adjunctive tool to anatomically and functionally assess lymphatic invasion. SPECT/CT improves the detection of sentinel nodes while decreasing operating time and improving cosmetic outcomes. 18F-fluorodeoxyglucose (18F-FDG) positron emission tomography/computed tomography (PET/CT) with small voxel reconstruction demonstrated increased specificity and sensitivity for detecting in-transit metastases of melanomas, specifically in the limbs. Dermoscopy allows providers to cost-effectively recognize common lesion patterns. Multiphoton microscopy assigns a weight-based score based on malignant features. Optical coherence angiography captures images of vessels to help diagnose equivocal lesions. Utilization of imaging techniques may increase diagnostic accuracy, reduce unnecessary procedures, and help guide treatment plans. Additional research is needed to further characterize the utility of these techniques in order to improve the diagnosis and treatment of melanomas.

## Introduction and background

Melanomas are one of the most treatment-resistant and aggressive cancers present in the human population [1]. It is the third most common cutaneous malignancy [2]. Each year in the United States, it is estimated that about 100,000 new cases of melanomas are diagnosed [2]. This cancer occurs due to the malignant transformation of the neural crest-derived cell, the melanocyte [1]. Typically, patients with a fair complexion and those who are more easily sunburned are predisposed to melanoma. However, family history, total sun exposure (UVA and UVB) over a lifetime, and the presence of atypical moles also contribute to melanoma etiology [1].

Melanomas may either arise in or near a precursor lesion or may arise de novo. Common precursor lesions include common acquired nevi, dysplastic nevi, congenital nevi, and cellular blue nevi. Melanomas can undergo two types of growth phases: radial and vertical. Malignant cells proliferate in the epidermis in a radial fashion during the radial phase and then progress to dermal invasion during the vertical growth phase. These lesions can be classified as thin (less than 1 mm), moderate (1-4 mm), and thick (greater than 4 mm). Melanomas are classified according to growth patterns: superficial spreading melanoma, nodular melanoma, lentigo maligna, and acral lentiginous melanoma [1]. Although melanomas mainly occur on the skin, metastasis can commonly occur in the lungs, liver, gastrointestinal tract, and brain due to invasion, angiogenesis, extravasation, dissemination, and colonization [2]. 

Patients are commonly diagnosed utilizing the “ABCDE '' acronym, that is, asymmetry, border regularity, color variations, diameter greater than 6 mm, and elevated surface. Typically, excisional biopsies are performed for diagnosis to allow for a proper histologic analysis [1]. In addition, the TNM system is utilized to categorize melanoma in five stages: 0, I, II, III, and IV. This system considers tumor thickness, ulceration, lymph nodal involvement, and metastasis. In situ melanoma is characterized as stage 0, while metastatic melanoma is stage IV [2]. 

Other imaging techniques, such as reflectance confocal microscopy, ultrasound (US), single-photon emission computed tomography/computed tomography (SPECT/CT), 18F-fluorodeoxyglucose (18F-FDG) positron emission tomography/computed tomography (PET/CT), dermoscopy, multiphoton microscopy, and optical coherence angiography can be used to diagnose and assess melanoma. These techniques are utilized to both assess primary lesions, such as lentigo maligna and melanoma lesions, and diagnose metastasis including lymph node and organ involvement. The aim of this scoping review is to identify, describe, and analyze imaging techniques implemented in the diagnosis of melanomas.

## Review

Methods

Search Strategy and Selection Criteria

A comprehensive literature search was performed on November 1, 2023, utilizing three databases: EMBASE, Medline, and PubMed. The systematic review utilized the Preferred Reporting Items for Systematic Reviews and Meta-Analyses (PRISMA) statement by the Cochrane Collaboration. Inclusion criteria stated that the articles must have been conducted in the human patient population in either a primary study, clinical trial, cohort study, or cross-sectional trial between January 1, 2013 to November 1, 2023. Articles were excluded if they were not in English, were review articles, or were conducted in animal populations. These criteria were selected to fulfill the objectives of this review. 

Key Terms

The key terms used to search for articles were as follows: dermatology, derm, skin lesions, skin imaging, confocal microscopy, laser microscopy, microscopy, optical coherence tomography, tomography, interferometry, ultrasound, Raman spectroscopy, spectroscopy, fluorescence imaging, dermoscopy, machine-based learning, machine learning, MBL, multispectral optoacoustic tomography, multispectral imaging, hyperspectral imaging, and multiphoton tomography. Databases were searched using the Boolean operator “AND” and “OR” as follows: (“dermatology*” OR (“derm*” OR “skin lesions*” OR “skin imaging*”)) AND (“confocal microscopy*” OR “laser microscopy*” OR “microscopy*” OR “optical coherence tomography*“ OR “tomography*” OR “interferometry*“ OR “ultrasound*” OR “Raman spectroscopy*“ OR “spectroscopy*” OR “fluorescence imaging*“ OR “dermoscopy*“ OR “machine-based learning*” OR “machine learning*“ OR “MBL*“ OR “multispectral optoacoustic tomography*” OR “multispectral imaging*“ OR “hyperspectral imaging*“ OR “multiphoton tomography*“)). These key terms were selected based on current practices utilized in dermatology. Articles were then further screened for melanoma-specific diagnostics. 

Evaluation Process 

**Figure 1:**
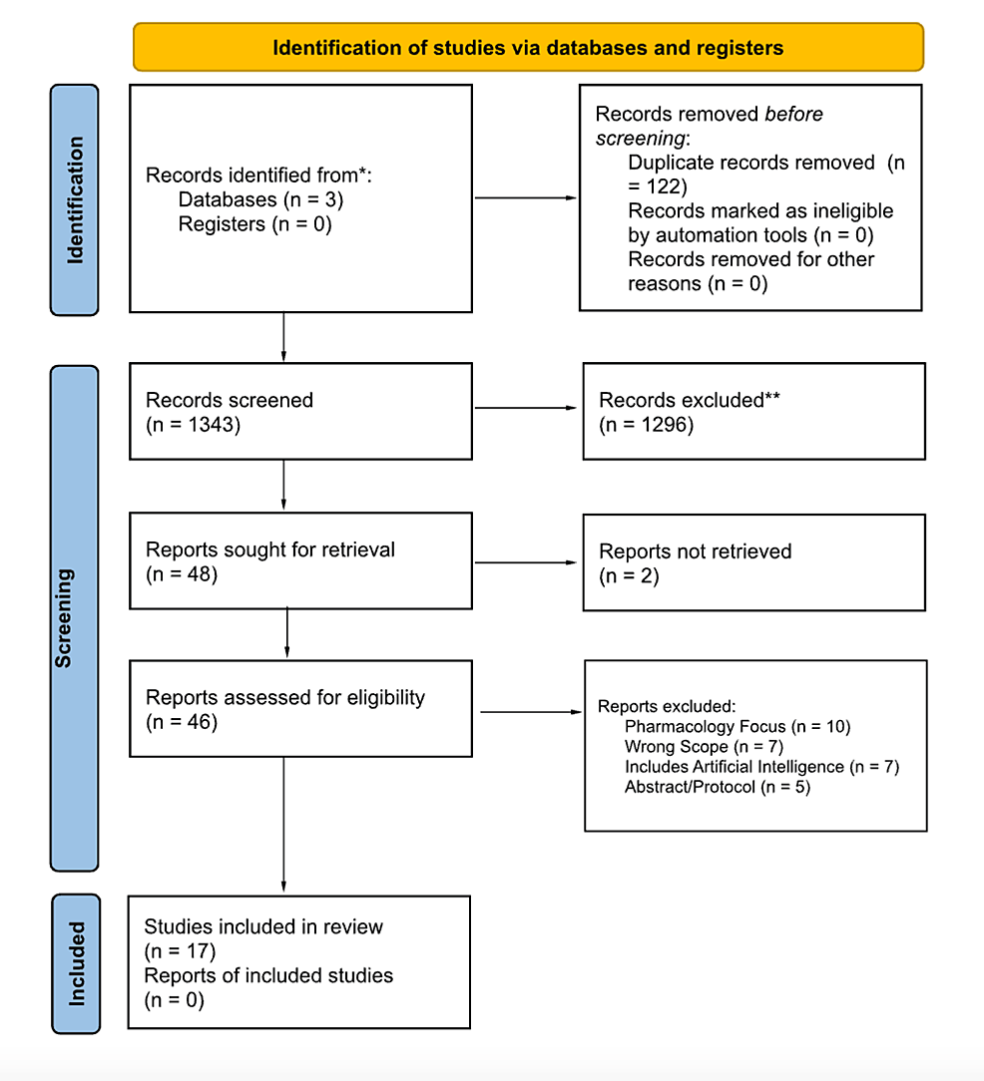
Preferred Reporting Items for Systematic Reviews and Meta-Analyses (PRISMA) diagram

The process of inclusion for the articles was portrayed in the PRISMA diagram (Figure 1). The authors (LS and MB) evaluated the same 1,343 articles after 122 duplicates were removed. Based on the inclusion and exclusion criteria, 1,296 articles were excluded and 48 were sought for retrieval. Forty-six were obtained and assessed for eligibility. The authors (LS, MB, HP, and CP) each independently reviewed the full-text publications. Due to further analysis, 10 were excluded due to a focus on pharmacology, seven articles were out of scope, seven articles utilized artificial intelligence, and five articles were conference abstracts or protocols. Disagreements were resolved by a discussion with all the aforementioned authors, yielding 17 total studies included in the review. Additional reference lists were not utilized. 

Data Collection 

The authors (LS and CP) independently evaluated each article and developed a data abstraction table, including author, publication year, study design, sample size, outcomes, limitations of the study, and recommendations to improve the study. Once data were synthesized and a qualitative analysis was performed on the table, the narrative review was written.

Results

RCM

RCM is a non-invasive imaging technique used to aid in the diagnosis of dermatologic lesions in vivo. This method helps provide high diagnostic accuracy and aids in the benign diagnosis of equivocal lesions [3]. Furthermore, RCM can be used to monitor dermatologic treatment outcomes and was found to have better sensitivity and specificity when compared to certain other imaging modalities. Adjunctive RCM decreases the number of unnecessary biopsies and excisions and assures the removal of aggressive melanoma with a Breslow thickness of 0.5 mm or thinner [3]. In a randomized controlled trial, RCM also had almost twice the positive predictive value (PPV) when compared to dermoscopy (18.9 vs. 33.3), a lower benign-to-malignant ratio, and resulted in a 43.4% reduction in the number needed to excise melanomas [3]. In addition, this study found that physicians’ years of RCM experience correlated highly with diagnostic accuracy. The use of RCM to more accurately diagnose melanomas should translate to reduced costs, shorter wait lists for surgery, and less delayed diagnoses [3].

RCM can be used to diagnose and assess the margins of lentigo maligna, a slow-growing subtype of melanoma. Imiquimod can be used in the treatment of lentigo maligna, and RCM was used to determine the lentigo maligna score (LMS) of lesions treated with imiquimod vs. those treated with placebo after one month of treatment [4]. This imaging modality also exhibited better sensitivity and specificity than multispectral digital skin lesion analysis (MDSLA) when comparing the efficacy in the detection of melanomas. RCM sensitivity and specificity were 85.7% and 66.7%, respectively, while MDSLA sensitivity and specificity were 71.4% and 25.0%, respectively [5]. This study found that RCM is useful as an adjunct imaging modality for suspicious melanocytic lesions and those requiring re-excision due to dysplasia or features of atypia. 

Ultrasound (US)

US is an imaging tool that is used in the diagnosis and treatment of a variety of medical conditions. This device transmits sound waves through a transducer to create images of structures inside the body and does not use any radiation. In the diagnosis of melanoma, US can be used to visualize lymph nodes and guide biopsies to determine metastasis. Ultrasound fine-needle aspiration cytology (US-FNAC) is a common practice in the diagnosis of many cancers including melanomas. Fifty percent of involved sentinel nodes can be identified using US-FNAC for melanoma patients [6]. Introduction of the Berlin US Morphology Criteria has shown improvement in up to 65-80% of patients as compared to the previous sensitivity of melanoma diagnosis of 20-40% [7]. In this study, US results were examined in reference to peripheral perfusion, loss of central echoes, and balloon shape. The presence of any of these three Berlin US Morphology Criteria prompted physicians to perform US-FNAC. Sentinel node biopsy (SNB) or lymphadenectomy was performed if the results were positive. The long-term survival of melanoma patients was found to be negatively correlated with positive US-FNAC results, so this imaging technique can be used to determine the need for further biopsy or excision. Positive biopsies conferred poor survival, so the patient could be spared SNB. SNB is recommended for those with suspicious US findings and negative FNAC to detect microscopic malignancy, while completely negative US-FNAC patients may choose to only follow up and avoid a biopsy altogether [7]. The presence of an echo-free island (EFI) is another diagnostic criterion used in US-FNAC. Of note, EFI had a 97.6% specificity in predicting malignant SN and was significantly associated with peripheral perfusion in 67.5% of cases. These data indicate that the presence of EFI in the US of a sentinel node was significantly associated with decreased five-year melanoma-specific survival, reporting 80% with EFI and 92% when absent. Therefore, EFI may be useful as an additional component of the Berlin US Morphology Criteria [6].

US elastography can also be used to differentiate between reactive and metastatic lymph nodes in the diagnosis of melanomas. Lymph node involvement is the most important prognostic factor for the survival of stage I and II melanoma patients, so SNBs are the current international recommendation. US elastography evaluates the elastic characteristics of structures by applying radiofrequency to the tissue to receive echo lines, compressing the tissue to cause displacement, and subsequent recording of post-compression echo lines on that tissue. Tissue density is then gathered from the elastogram, and pathological findings are determined. In this study, an experienced radiologist used a node-to-node correlation of the lymph node size and morphology [8]. Lymph nodes with higher percentages and distribution of high elasticity or hardness were considered to be malignant metastases. In addition, loss of central echoes and balloon shape were used to qualify the likelihood of malignancy using B-mode US evaluation. The accuracy of lymph node metastasis evaluation by B-mode and elastography with an elastography score (ES) cutoff of 3 were 70% and 90%, respectively. Evaluation of US elastography with an ES cutoff of 4 yielded 95% accuracy. The study findings indicate that a higher ES score is more associated with metastatic lymph nodes compared to reactive nodes. In addition, high elasticity was measured subjectively, and the simplicity of the ES score allows for reasonable diagnostic accuracy regardless of examiner experience. Therefore, the use of US elastography can enhance diagnostic accuracy and eliminate unnecessary SNBs.

SPECT/CT

SPECT/CT can be used to aid in the diagnosis of metastatic lymph node spread of melanoma. SNBs have a false negative rate of 44%, and performing SPECT/CT can provide complementary anatomical and functional information about the tissue. This imaging modality can increase the accuracy of the anatomic location, identify false positives from planar imaging, reduce the number of false-negative SNBs, and provide more specific information about the lesion to improve the surgical approach [8]. In this study, SPECT/CT helped correctly detect significantly more SN than the standard group (2.40 vs. 1.87; p < 0.001), and the number of positive SNB was significantly higher (0.34 vs. 0.21; p = 0.038). Removal of these positive SNs leads to improvement of life years. In addition, SPECT/CT permits smaller incisions, allowing for reduced operating time and the use of local anesthesia. This results in better cosmetic results with a greatly reduced cost of surgery [9].

18F-FDG PET/CT

Melanoma is an aggressive cancer that requires accurate staging for proper diagnosis and management. 18F-FDG PET/CT scans can detect soft tissue, nodal, and visceral metastases with a high diagnostic accuracy. Patients are injected with radioactive dye and the area of interest is scanned, followed by a low-dose unenhanced CT scan to improve localization and provide attenuation correction. With the help of an experienced PET reader, Zimmermann et al. were able to determine the presence of in-transit metastases of melanoma to the limbs in patients with biopsy-proven melanoma [10]. The reader was only aware of the location of the primary tumor and any previous biopsies or surgeries to reduce the number of false positives. The use of 18F-FDG PET/CT resulted in less indeterminate and less false-negative results in European Association of Nuclear Medicine Research Limited (EARL)-compliant lesions using 1 mm point-spread function (PSF) small voxel reconstruction. Seven out of 32 EARL-compliant images moved from negative to positive, and five out of six images classified as indeterminate were moved to positive on 1 mm PSF images (p = 0.01). The results were confirmed by pathology, clinical, and follow-up imaging. The use of thin-matrix reconstruction (1 mm) resulted in increased sensitivity and specificity of 92% and 94%, respectively, compared to 73% and 91% for EARL-complaint reconstruction [10]. In addition, negative likelihood ratios for these imaging parameters were almost perfect. These results support the use of small voxel reconstruction in 18F-FDG PET/CT imaging studies in the detection of in-transit melanoma metastases, specifically in the limbs [10]. This technology can reduce indeterminate findings and compensate for false-negative scans in older PET systems.

Dermoscopy

Dermoscopy is a non-invasive magnifying tool that can be used to diagnose melanocytic lesions among other dermatologic lesions based on characteristics not visible to the naked eye. For melanoma, optical dermoscopy improves diagnostic sensitivity for melanoma with a 90% sensitivity vs. 74% with the naked eye [11]. Digital dermoscopy was found to have a specificity of 84% in the detection of melanoma among atypical melanocytic lesions and helps reduce the number of unnecessary excisions when compared to clinical diagnosis with the naked eye and optical dermoscopy [12]. Common dermoscopic patterns when examining melanoma include asymmetry, blotches, multicolor patterns, blue-white veils, atypical pigment networks, irregular peripheral streaks, atypical vascular patterns, ulcers, atypical dots/globules, white shiny lines, brown peripheral structureless areas, and regression structures. Among these features, asymmetry and multicolor patterns showed up in the majority of melanomas [11]. In addition, a study of 150 melanocytic lesions also found that a negative pigment network and radial streaming or pseudopods were highly specific for higher-grade lesions [13]. Lesions were graded based on increasing levels of atypia from none to invasive melanoma. Of note, a negative pigment network and radial streaming or pseudopods had 95.8% specificity for an upgrade. When diagnosing melanoma via dermoscopy, a number of scores can be obtained including the seven-point checklist, revised seven-point checklist, three-point checklist, ABCD rule, and CASH algorithm. Melanoma-related dermoscopic patterns were found to be common in Asian patients on non-acral surfaces, and it is therefore a reliable diagnostic tool [11]. 

The introduction of dermoscopy into primary care can also reduce the number of unnecessary excisions and referrals to secondary care. In a study of 48 Dutch practices, 194 lesions were examined and results showed that the probability of correct diagnosis was 1.25x higher with dermoscopy (p = 0.07). These data indicate that dermoscopy appears to be a cost-effective way to improve dermatologic diagnosis in the primary care setting, and there is reason for primary care providers to be trained to help evaluate the need for excision or secondary care referral [14]. 

It is important to note that all lesions suspected of being melanomas on dermoscopy must then be analyzed via microscopy for definitive diagnosis. However, histopathologic assessment can be influenced by inter-rater and intra-rater variability. Histology only captures a certain cross-section of the lesion at one point in time, and features of severely dysplastic nevi and melanomas can overlap significantly. Dermoscopy can help guide diagnosis by pinpointing the optimal plane for sectioning and providing additional assessment factors in vivo. In a study of 136 cases with and without dermoscopy, the addition of dermoscopy led to changes in lesion grading in 33/136 (24.3%), with 16 cases being upgraded to melanoma only after the addition of dermoscopy [15]. Fleiss’ kappa, a statistical test of inter-rater agreement, increased from 0.447 to 0.496 with dermoscopy across all cases in the study. These data support the notion that dermoscopy improves inter-observer agreement in histopathologists in the diagnosis of pigmented lesions [15].

Dermoscopy may also be used to diagnose second primary melanomas (SPMs) induced by drugs, such as vemurafenib [12]. In this context, the most common features were color changes (up to 15%) and the appearance/disappearance of globules (14.6%) over a mean follow-up duration of 6.7 months. Digital dermoscopy allowed physicians to only excise 36/2155 lesions examined and detect 14 SPMs. The benign-to-malignant ratio was 63.6% [12]. 

Super-high (400x) magnification dermoscopy (D400) has also recently been developed and can identify additional dermatologic features including single melanocytes. With D400, physicians can identify specific pigmented cells and their morphology with p-values of 0.001-0.01 that cannot be visualized using traditional dermoscopy (D20). The super-high magnification allows for the comparison of the presence of these features in malignant melanoma vs. nevi [16]. 

Dermoscopic features may also overlap in lesions with similar pathologic origin. A percentage (38.7%) of patients with multiple primary melanomas were found to have similar dermoscopic features, and these melanomas showed 5.7x higher odds to share the same BRAF mutational status [17]. However, dermoscopy, like all imaging techniques, has its limitations. Dermoscopy was not found to show any significant differences in BRAF V600E vs. V600K mutations in malignant melanoma [18]. These results emphasize the importance of molecular analysis in determining the specifics of the malignancy and how to properly manage patients. 

Multiphoton Microscopy (MPM) and Optical Coherence Angiography (OCA)

A wide range of melanocytic lesions may be equivocal, and therefore indistinguishable, from melanoma on visual and dermoscopic exams. Combined use of multiphoton microscopy (MPM) and optical coherence angiography (OCA) can aid in the differential diagnosis of these lesions [19]. MPM consists of a femtosecond laser, a near-infrared optics arm, and a beam-scanning module. The tissue is excited, producing fluorescence in the epidermis and a collagen signal in the dermis. Sections are then acquired and lesions are assigned an MPM score (MPMS), which is a sum of the malignant features multiplied by a weight factor. The malignant features include polymorphic cells, dendritic structures, and pagetoid cells, and a higher score was associated with melanoma. OCA involves a laser and an articulating probe that contacts the skin and captures real-time images of the angiographic contrast. The images are filtered to reduce artifacts, and then the vasculature is described by dermatologists using dermoscopic terminology. Vessel density and length were calculated and documented, and densely packed vessels with large, irregularly arborizing vessels were indicative of melanoma. Specifically, invasive melanoma had thicker vessels and higher vessel density than melanoma in situ and benign lesions. Of the 60 lesions that were clinically and dermoscopically evaluated, 18 were considered difficult to diagnose. By using MPM and OCA, 12 out of 18 of these lesions were found to be melanomas of varying infiltration and type. In addition, all equivocal lesions were able to be diagnosed correctly using discriminant function analysis. Therefore, the combined use of MPM and OCA may play a role in improving the early diagnosis of melanoma [19]. Table 1 details the results of each study. 

**Table 1:** Results of the studies MM: malignant melanoma, MPM: multiphoton microscopy, OCA: optical coherence angiography, BRAF: serine/threonine-protein kinase B-Raf, CMM: cutaneous malignant melanoma, ES: elastography score, US-FNAC: ultrasound-guided fine-needle aspiration cytology, US: ultrasound, FNAC: fine-needle aspiration cytology, RCM: reflectance confocal microscopy, SPM: second primary melanoma, MDSLA: multispectral digital skin lesion analysis, SPECT/CT: single-photon emission computed tomography/computed tomography, EFI: echo-free island, PPV: positive predictive value, NPV: negative predictive value, PET/CT: positron emission tomography and computed tomography, 18F-FDG: fludeoxyglucose F18, EAR: European Association of Nuclear Medicine Research Limited

Reference	Study type	Sample size	Aim	Findings	Limitations	Recommendations
Cinotti et al., Super-high magnification dermoscopy can aid the differential diagnosis between melanoma and atypical naevi. Clin Exp Dermatol. 2021 Oct; 46(7):1216-1222 [16]	Prospective observational	79 patients	Assess the usefulness of super-high (400x) dermoscopy compared to standard (20x) dermoscopy in the diagnosis of malignant melanoma (MM)	Super-high (400X) dermoscopy was able to identify morphologic features that standard (20x) dermoscopy was unable to identify, which could be useful in the diagnosis of MM.	Sample size	Consider performing a randomized trial to evaluate the significance of the findings
Elagin et al., In vivo multimodal optical imaging of dermoscopic equivocal melanocytic skin lesions. Sci Rep. 2021 Jan 14;11(1):1405 [19]	In vivo qualitative analysis.	60 melanocytic lesions.	Determine if Multiphoton Microscopy (MPM) and Optical Coherence Angiography (OCA) can be useful positive/negative predictors for melanocytic lesions.	MPM can differentiate benign versus melanocytic lesions and OCA can differentiate invasive and non-invasive lesions.	Sample size.	Reproduce results with larger sample size across multiple healthcare systems.
Koelink et al. Diagnostic accuracy and cost-effectiveness of dermoscopy in primary care: a cluster randomized clinical trial. J Eur Acad Dermatol Venereol. 2014 Nov;28(11):1442-9 [14]	Randomized Controlled Trial.	416 lesions, 381 patients.	Determine the benefits of using dermoscopy in primary care.	Dermoscopy improves sensitivity for diagnosing melanoma up to 15% in primary care, probability of correct diagnosis is 1.25x higher but not statistically significant. Dermoscopy may reduce unnecessary referrals and biopsies and is cost-effective.	May be less applicable in the United States due to the workflow of primary care physicians.	Broaden the trial to the continental US to assess the reliability of the study.
Merkel et al. The utility of dermoscopy-guided histologic sectioning for the diagnosis of melanocytic lesions: A case-control study. Journal of the American Academy of Dermatology, 2016. 74(6), 1107–1113 [13]	Case-control.	150 melanocytic lesions.	Evaluate the diagnostic utility of dermoscopy-guided micropunch score in the evaluation of melanocytic lesions.	Dermoscopy was found to provide clinically significant information that was used to optimize the sectioning of melanocytic lesions. The authors found that regression structures, radial streaming, and irregular blotches were highly specific for a higher diagnostic grade.	Retrospective nature of case-controlled studies. Non-authentic sectioning of lesions.	Investigate the utility of the protocol in the primary analysis of melanocytic lesions in an authentic clinical setting.
Moscarella et al. Dermoscopic similarity is an independent predictor of BRAF mutational concordance in multiple melanomas. Exp Dermatol. 2019 Jul;28(7):829-835 [17]	Prospective.	124 melanomas, 62 patients.	Assess if patients with BRAF mutations exhibit dermoscopically similar melanomas.	Dermoscopically similar lesions are a positive indicator for BRAF mutations in Multiple Primary Melanoma patients.	Inherent study design lacks controlled comparisons.	Consider adjusting the study type to more rigorously assess correlation.
Mun et al. Dermoscopy of Melanomas on the Trunk and Extremities in Asians. PLoS One. 2016 Jul 8;11(7):e0158374 [11]	In vivo qualitative analysis.	22 melanocytic lesions.	Investigate the dermatoscopic patterns of melanocytic lesions on the trunk/extremities of the Asian population.	Dermoscopy can be useful in characterizing malignant melanoma among specific populations and anatomical locations.	Sample size.	Recreate study for multiple ethnicities/nationalities to characterize malignant melanoma characteristics.
Ogata et al. Accuracy of real-time ultrasound elastography in the differential diagnosis of lymph nodes in cutaneous malignant melanoma (CMM): a pilot study. Int J Clin Oncol. 2014 Aug;19(4):716-21 [8]	Prospective.	12 patients, 20 lymph nodes.	Assess the diagnostic ability of real-time elastography to differentiate between reactive and metastatic Lymph Nodes in cutaneous malignant melanoma (CMM) and to determine the optimal cut-off value to diagnose CMM.	The sensitivity, specificity, and accuracy of elastography were greater than that of B-mode ultrasound with respect to diagnosing metastatic LNs, both with an Elastography Score (ES) cutoff of 3 and 4. The optimal ES cutoff was deemed to be greater than or equal to 4.	Operator dependent. Small sample size. Subjective scoring system.	Consider repeating the study with a larger cohort and attempting to objectively quantify Elastography scores.
Oude et al. Long-term results of ultrasound guided fine needle aspiration cytology in conjunction with sentinel node biopsy support step-wise approach in melanoma. Eur J Surg Oncol. 2017 Aug;43(8):1509-1516 [6]	Cohort.	1,000 patients.	Determine the effectiveness of Ultrasound-guided Fine Needle Aspiration Cytology (US-FNAC) regarding Melanoma Specific Survival (MSS).	Positive US-FNAC patients had worse survival rates compared to those with normal US findings. Patients with normal US findings had comparable survival rates to those with suspicious US findings and a negative FNAC.	Results could diverge without expert ultrasonographers, as seen in this study.	Consider outlining the sonographic approach and repeating the study with a variety of US technicians.
Pellacani et al. Effect of Reflectance Confocal Microscopy for Suspect Lesions on Diagnostic Accuracy in Melanoma: A Randomized Clinical Trial. JAMA Dermatol. 2022 Jul 1;158(7):754-761. doi: 10.1001/jamadermatol.2022.1570. Erratum in: JAMA Dermatol. 2023 May 1;159(5):566 [3]	Randomized Controlled Trial.	3,165 patients.	Determine if Reflectance Confocal Microscopy (RCM) reduces unnecessary excision rates by 30% and determine if RCM identifies all melanoma lesions thicker than 0.5 mm.	Adjunctive RCM for suspect lesions decreases unnecessary excisions and assures the removal of aggressive melanomas thicker than 0.5mm in a real-world scenario.	Minor reduction in reliability due to loss of follow-up/refusal of excision.	Determine characteristics that allow melanoma lesions less than 0.5mm thick to be unreliably diagnosed.
Perier-Muzet al. Melanoma patients under vemurafenib: prospective follow-up of melanocytic lesions by digital dermoscopy. J Invest Dermatol. 2014 May;134(5):1351-1358 [12]	Prospective.	2,155 melanocytic lesions.	Characterize the dermatoscopic signs of second primary melanomas (SPMs) and changes of other documented skin lesions in patients taking Vemurafenib for BRAF V600E mutations.	56.1% of documented skin lesions exhibited dermatoscopic change.	Patients lost to follow-up, sample size decreased.	Reproduce findings with different technicians and patient populations to test patterns of change and reliability of change.
Shi et al. Incorporation of dermoscopy improves inter-observer agreement among dermatopathologists in histologic assessment of melanocytic neoplasms. Arch Dermatol Res. 2021 Mar;313(2):101-108 [15]	Prospective cohort.	136 patients.	Assess whether the addition of dermoscopy to routine histopathologic analysis improves inter-observer reliability for the diagnosis of melanocytic lesions.	Inter-rater reliability with Fleiss’ kappa statistic showed an increase from 0.447 without dermoscopy to 0.496 with the addition of dermoscopy. The total atypia grade changed in 24.3% of cases after the addition of dermoscopy.	Sample size. Observer/expert size. Came from a clinic of high-risk patients so results may be more profound than the general population.	Consider retrospective analysis of erroneously diagnosed melanocytic lesions with the addition of dermoscopy to routine histopathologic analysis to assess true clinical benefit.
Soenen et al. Change in lentigo maligna score assessed by in vivo reflectance confocal microscopy after 1 month of imiquimod treatment for lentigo maligna management. J Am Acad Dermatol. 2022 May;86(5):1042-1048 [4]	Randomized Controlled Trial.	40 patients.	Assess whether Imiquimod therapy aids in the treatment of Lentigo Maligna via Reflectance Confocal Microscopy.	The Lentigo Maligna Score was significantly less in those treated with Imiquimod.	Small sample size.	Utilize Reflectance Confocal Microscopy to monitor the treatment response of immunomodulators to other melanocytic lesions.
Song et al. Paired comparison of the sensitivity and specificity of multispectral digital skin lesion analysis and reflectance confocal microscopy in the detection of melanoma in vivo: A cross-sectional study. J Am Acad Dermatol. 2016 Dec;75(6):1187-1192.e2 [5]	Cross-sectional.	36 patients, 55 lesions.	Compare the practicality of Multispectral Digital Skin Lesion Analysis (MDSLA) vs Reflectance Confocal Microscopy (RCM) in the detection of melanoma.	RCM sensitivity and specificity were 85.7% and 66.7%, respectively. MDSLA sensitivity and specificity was 71.4% and 25.0%, respectively. Both modalities can be costly, time-consuming, and operator-dependent. RCM may be more appropriate as an adjunctive diagnostic tool.	Small sample size from one dermatologist’s office.	Repeat the study with a more diverse population.
Stoffels et al. Sentinel lymph node excision with or without preoperative hybrid single-photon emission computed tomography/computed tomography (SPECT/CT) in melanoma: study protocol for a multicentric randomized controlled trial. Trials. 2019 Feb 4;20(1):99 [9]	Randomized controlled trial.	Preliminary data includes 402 patients.	Protocol aimed at determining the effectiveness of metastasis-free survival of SPECT/CT in completing sentinel node excision for melanomas.	SPECT/CT aided Sentinel Lymph Node Excision improves 5-year metastasis-free survival.	Preliminary data.	Not applicable, currently.
Voit et al. Ultrasound of the sentinel node in melanoma patients: echo-free island is a discriminatory morphologic feature for node positivity. Melanoma Research 26(3):p 267-271, June 2016 [6]	Prospective database	1000 patients.	Examine a new criterion, EFI used in ultrasound in regards to sentinel node diagnosis in melanoma	EFI was seen in 4% of patients, sensitivity and specificity were 10.8% and 97.6% respectively, PPV was 50%, and NPV was 80.2%. There is a significant correlation between EFI and peripheral perfusion. Melanoma-specific survival over 5 years was significantly worse with the presence of EFI	EFI occurrence is very rare, and US accuracy is very user dependent	Further research may be done to determine the etiology behind EFI and how it may be related to other prognostic indicators in melanoma diagnosis and management
Zengarini et al. BRAF V600K vs. BRAF V600E: a comparison of clinical and dermoscopic characteristics and response to immunotherapies and targeted therapies. Clin Exp Dermatol. 2022 Jun;47(6):1131-1136 [18]	Retrospective.	132 patients.	Compare the clinical, dermatoscopic, and prognostic features of BRAF V600E MMs and BRAF V600K MMs.	No clinical nor dermatoscopic features were able to reliably delineate between the different mutations. BRAF V600K mutations were associated with earlier metastasis and poorer response to therapy.	Small sample size, inherent limitations of retrospective studies.	Repeat the trial with a larger, prospective cohort.
Zimmermann et al. Revisiting detection of in-transit metastases in melanoma patients using digital 18F-FDG PET/CT with small-voxel reconstruction. Ann Nucl Med. 2021 Jun;35(6):669-679 [10]	Retrospective.	46 images.	Compare the effectiveness of identifying in-transit malignant melanoma (MM) metastases from the extremities with ^18^F-FDG PET/CT with small voxels reconstruction compared to standard reconstruction with European Association of Nuclear Medicine Research Limited (EARL) images.	Digital PET/CT with small voxel reconstruction (1mm_PSF_) changed 12 images from negative/intermediate to positive for in-transit metastases. Overall, the positive likelihood ratio of small-voxel reconstruction was 14.7, compared to standard reconstruction with EARL at 7.82.	Sample size, selection bias.	Compare PET/CT reconstructions in a randomized controlled trial to determine the clinical utility of small voxel reconstruction.

Discussion 

Melanomas are one of the most aggressive cancers that occur from the malignant transformation of the neural crest-derived cell known as the melanocyte [1]. Melanoma risk factors include but are not limited to fair complexion, family history, overall sun exposure (UVA and UVB), and the presence of atypical moles [1]. 

There are many common precursor lesions, such as common acquired nevi, dysplastic nevi, congenital nevi, cellular blue nevi, and dysplastic nevi, which are further categorized by size, growth pattern, and presence of metastases [2]. About one-third of these precursor lesions may transform into malignant melanomas [13]. 

Further melanoma is diagnosed with the "ABCDE'' acronym and the TNM system, but imaging techniques can be useful in diagnosis. The more common imaging techniques include RCM, US, SPECT/CT, 18F-FDG PET/CT, dermoscopy, MPM, OCA, traditional microscopy, and whole-slide imaging. 

Imaging Modalities 

RCM accurately diagnoses equivocal lesions and can be useful to evaluate the influence of treatments used [3]. Similarly, MPM and OCA can be used to aid in diagnosing equivocal lesions as well. Specifically, adjunctive RCM reduces the number of biopsies and effectively identifies aggressive melanoma with a Breslow thickness of 0.5 mm or less [3]. Overall, RCM has superior sensitivity and specificity compared to other imaging modalities. RCM also has a greater PPV, lower benign-to-malignant ratio, and greater diagnostic accuracy when compared to dermoscopy and MDSLA [3,5]. 

Although the PPV for RCM was found to be double the PPV of dermoscopy [3], optical dermoscopy demonstrates 90% diagnostic sensitivity for melanoma while RCM has a pooled sensitivity of 92%. Digital dermoscopy was found to have an 84% specificity when evaluating atypical melanocytic lesions, which is greater than the specificity values of optical dermoscopy and the naked eye [12]. An advantage of dermoscopy is the ease of use for primary care physicians, which can contribute to early diagnosis and treatment, ultimately enhancing the life expectancy of patients. While microscopy provides more in-depth analysis, using dermoscopy before microscopy determines the relevant sections to focus on, which can prevent potential melanoma diagnoses from being overlooked. In the study conducted by Shi et al., the addition of dermoscopy changed lesion grading in 33/136 cases (24.3%). Of the 33 cases, 16 were determined to be melanoma (48%) [15]. This finding significantly impacts the treatment approach and survival rate in a patient. 

To investigate lesion malignancy, MPM and OCA are potential imaging modalities. MPM utilizes the MPMS, which is a sum of malignant features in a cross-sectional area, aiding in distinguishing if the lesion is malignant or benign. OCA captures images, which can help clinicians visualize certain lesions. In the study conducted by Elagin et al., 18 out of the 60 lesions evaluated were difficult to diagnose [19]. When MPM and OCA were used in conjugation, 12 out of the 18 lesions were found to be melanomas. The ability to further identify lesions that at first were considered equivocal helps to improve survival outcomes because early diagnosis can be made and the correct stage can be determined. In relation to RCM, MPM and OCA reduce the need for biopsies.

Furthermore, US-FNAC is an imaging modality used to diagnose many cancers, such as melanoma. The results of the study conducted by Oude et al. suggested a negative correlation between positive US-FNAC results and long-term survival with melanoma [7]. This finding indicates that positive biopsy results align with poor survival, eliminating further SNB, and avoiding more invasive procedures. Thus, US-FNAC shows potential to be used as an imaging technique to determine if further biopsy or excision is needed. 

Another technique that helps differentiate between reactive and metastatic lymph nodes in melanoma is US elastography, which uses elastic characteristics, radiofrequency, and the compression of tissues to determine pathology [8]. This method has a consistent elastography score system, which minimizes diagnosis errors that are dependent on the experience of the technician. A lymph node with high elasticity was considered malignant, and when there was a loss of central echoes and a balloon shape, the likelihood of malignancy was identified [8]. Similarly, SPECT/CT and 18F-FDG PET/CT are useful imaging modalities to help diagnose the metastatic lymph node spread of melanoma. SPECT/CT can provide greater anatomical and functional information about tissues, which can help identify false positives and reduce false-negative SNBs [9]. In addition, smaller incisions are needed in SPECT/CT, which makes it a favorable imaging technique because less scarring is present. Furthermore, 18F-FDG PET/CT scans are useful in detecting tissue and visceral metastases, especially in limb metastases, providing superior specificity and sensitivity compared to other imaging modalities.

*Limitations* 

Limitations that should be considered when interpreting the results and implications of this study include that literature on imaging techniques may have been potentially missed. Although this was likely decreased due to the search that was conducted on multiple databases, it is still possible. Other literature may have been excluded due to the limitation of the search for English-language publications only. Several of the studies also had small sample sizes and some patients were lost to follow-up, making the interpretation of the results difficult. In addition, searching for the literature from 2013 onwards may have excluded some prior studies in melanoma diagnosis; however, this ensures that the data presented are the most up-to-date. Other terms that could have been included to broaden the search may include “Breslow thickness,” “lentigo maligna,” “acral lentiginous melanoma,” and “nodular melanoma.” Expanding the search criteria may produce other high-quality literature. Lastly, limiting the literature search to peer-reviewed articles ensured high-quality data collection. 

Future Studies 

Future studies should focus on obtaining a greater sample size to replicate results that are similar in clinical practices. Furthermore, skin cancers are common, and there are many types, such as squamous cell carcinoma and basal cell carcinoma, so performing studies evaluating other skin malignancies is important to further increase diagnostic accuracy and life expectancy. In addition, skin complexion is an important factor to consider in evaluating skin lesions because malignant lesions vary in appearance. Future studies should focus on how to properly assess lesions in a variety of skin complexions and how the subsequent diagnosis and use of imaging modality can differ to properly educate physicians. Lastly, US results vary depending on technicians. For each study, having a consistent technician evaluate and monitor the lesions at hand can improve the sensitivity of the results.

## Conclusions

This scoping review describes different imaging studies that are used to diagnose malignant melanomas. The diagnosis of melanomas may include various studies to determine the stage of the tumor. These studies help inform the management of the cancer. This manuscript explains how the utilization of imaging techniques may have a positive impact on diagnostic accuracy, reduce unnecessary procedures, and help guide treatment plans. More research is warranted on these techniques to help healthcare professionals improve the diagnosis and treatment of melanomas. Ideally, these studies will include a large sample size of diverse participants and results will be interpreted by several physicians to maximize accuracy and reliability. These techniques may also be applied to other dermatologic conditions and medical problems.

## References

[1] Heistein J, Acharya U, S S (2023). Malignant melanoma. StatPearls [Internet].

[2] Sundararajan S, Thida A, Yadlapati S (2023). Metastatic melanoma. StatPearls [Internet].

[3] Pellacani G, Farnetani F, Ciardo S (2022). Effect of reflectance confocal microscopy for suspect lesions on diagnostic accuracy in melanoma: a randomized clinical trial. JAMA Dermatol.

[4] Soenen A, Vourc'h M, Khammari A (2022). Change in lentigo maligna score assessed by in vivo reflectance confocal microscopy after 1 month of imiquimod treatment for lentigo maligna management. J Am Acad Dermatol.

[5] Song E, Grant-Kels JM, Swede H (2016). Paired comparison of the sensitivity and specificity of multispectral digital skin lesion analysis and reflectance confocal microscopy in the detection of melanoma in vivo: a cross-sectional study. J Am Acad Dermatol.

[6] Voit CA, Oude Ophuis CM, Ulrich J, van Akkooi AC, Eggermont AM (2016). Ultrasound of the sentinel node in melanoma patients: echo-free island is a discriminatory morphologic feature for node positivity. Melanoma Res.

[7] Oude Ophuis CM, Verhoef C, Grünhagen DJ (2017). Long-term results of ultrasound guided fine needle aspiration cytology in conjunction with sentinel node biopsy support step-wise approach in melanoma. Eur J Surg Oncol.

[8] Ogata D, Uematsu T, Yoshikawa S, Kiyohara Y (2014). Accuracy of real-time ultrasound elastography in the differential diagnosis of lymph nodes in cutaneous malignant melanoma (CMM): a pilot study. Int J Clin Oncol.

[9] Stoffels I, Herrmann K, Rekowski J, Jansen P, Schadendorf D, Stang A, Klode J (2019). Sentinel lymph node excision with or without preoperative hybrid single-photon emission computed tomography/computed tomography (SPECT/CT) in melanoma: study protocol for a multicentric randomized controlled trial. Trials.

[10] Zimmermann PA, Houdu B, Césaire L, Nakouri I, De Pontville M, Lasnon C, Aide N (2021). Revisiting detection of in-transit metastases in melanoma patients using digital (18)F-FDG PET/CT with small-voxel reconstruction. Ann Nucl Med.

[11] Mun JH, Ohn J, Kim WI, Park SM, Kim MB (2016). Dermoscopy of melanomas on the trunk and extremities in Asians. PLoS One.

[12] Perier-Muzet M, Thomas L, Poulalhon N (2014). Melanoma patients under vemurafenib: prospective follow-up of melanocytic lesions by digital dermoscopy. J Invest Dermatol.

[13] Merkel EA, Amin SM, Lee CY (2016). The utility of dermoscopy-guided histologic sectioning for the diagnosis of melanocytic lesions: a case-control study. J Am Acad Dermatol.

[14] Koelink CJ, Vermeulen KM, Kollen BJ, de Bock GH, Dekker JH, Jonkman MF, van der Heide WK (2014). Diagnostic accuracy and cost-effectiveness of dermoscopy in primary care: a cluster randomized clinical trial. J Eur Acad Dermatol Venereol.

[15] Shi K, Compres E, Walton KE (2021). Incorporation of dermoscopy improves inter-observer agreement among dermatopathologists in histologic assessment of melanocytic neoplasms. Arch Dermatol Res.

[16] Cinotti E, Tognetti L, Campoli M (2021). Super-high magnification dermoscopy can aid the differential diagnosis between melanoma and atypical naevi. Clin Exp Dermatol.

[17] Moscarella E, Pellegrini C, Pampena R (2019). Dermoscopic similarity is an independent predictor of BRAF mutational concordance in multiple melanomas. Exp Dermatol.

[18] Zengarini C, Mussi M, Veronesi G, Alessandrini A, Lambertini M, Dika E (2022). BRAF V600K vs. BRAF V600E: a comparison of clinical and dermoscopic characteristics and response to immunotherapies and targeted therapies. Clin Exp Dermatol.

[19] Elagin V, Gubarkova E, Garanina O (2021). In vivo multimodal optical imaging of dermoscopic equivocal melanocytic skin lesions. Sci Rep.

